# Automated Evaluation of *Crithidia luciliae* Based Indirect Immunofluorescence Tests: A Novel Application of the EUROPattern-Suite Technology

**DOI:** 10.1155/2015/742402

**Published:** 2015-10-25

**Authors:** Stefan Gerlach, Kai Affeldt, Lena Pototzki, Christopher Krause, Jörn Voigt, Johanna Fraune, Kai Fechner

**Affiliations:** Institute for Experimental Immunology, EUROIMMUN, Seekamp 31, 23560 Lübeck, Germany

## Abstract

Systemic lupus erythematosus (SLE) is a severe rheumatic autoimmune disease with various clinical manifestations. Anti-dsDNA antibodies are an important immunological hallmark of SLE and their occurrence represents a major criterion for the diagnosis. Among the commonly applied test systems for determination of anti-dsDNA antibodies, the indirect immunofluorescence test (IIFT) using the flagellated *kinetoplastida Crithidia luciliae* is considered to be highly disease specific at moderate sensitivity. Since IIFT, however, is claimed to be affected by subjective interpretation and a lack of standardization, there has been an increasing demand for automated pattern interpretation of immunofluorescence reactions in recent years. Corresponding platforms are already available for evaluation of anti-nuclear antibody (ANA) IIFT on HEp-2 cells, the recommended “gold standard” for ANA screening in the diagnosis of various systemic rheumatic autoimmune diseases. For one of these systems, the “EUROPattern-Suite” computer-aided immunofluorescence microscopy (CAIFM), automated interpretation of microscopic fluorescence patterns was extended to the *Crithidia luciliae* based anti-dsDNA IIFT.

## 1. Introduction

For diagnosis of systemic lupus erythematosus (SLE), determination of autoantibodies is of significant diagnostic importance [1, 2]. Among these, antibodies against double-stranded DNA (anti-dsDNA) play a major role. Their presence constitutes an important immunological criterion for the diagnosis of SLE as stated by the American College of Rheumatology in 1982 [3]. A more recent approach by the Systemic Lupus Collaborating Clinics to revise and validate the American College of Rheumatology SLE classification criteria approved anti-dsDNA as a major serological feature of SLE, considering them as very specific and a marker of disease activity and kidney involvement [4]. Accordingly, studies in mice and humans provided evidences for a role of anti-dsDNA in the pathogenesis of lupus nephritis [2, 5–9].

Information on the prevalence of anti-dsDNA in SLE varies between studies, ranging from 30% to 98% [2, 10]. The application of different laboratory tests is one cause which contributes to this deviation [11, 12]. The most common methods for the detection of anti-dsDNA are enzyme-linked immunosorbent assays (ELISA), radio immunoassays (RIA, e.g., Farr assays and PEG assays) and* Crithidia luciliae* indirect immunofluorescence tests (CLIFT) [13]. It is hypothesized that each of these detects individual, yet overlapping, subgroups of anti-dsDNA revealing divergent properties (e.g., avidity, structural specificity) and, of particular interest, different clinical associations [10, 14]. Classical anti-dsDNA ELISA is accepted as the most sensitive but often less specific method for SLE diagnostics. Through modifications of the applied DNA substrates and their linkage to the test wells, an increase in diagnostic accuracy of the ELISA for SLE could be achieved in recent years [15, 16]. Nevertheless, consistency between different ELISA kits seems to be limited [12]. Therefore, primary test results usually require confirmation by a second assay such as Farr immunoassay and/or CLIFT, both of which are regarded as highly disease specific, detecting only antibody subpopulations with a high positive predictive value for SLE [10, 12, 14, 17–19]. Since RIA employ radioactive elements, CLIFT is commonly considered as more applicable confirmatory test system in the clinical routine of SLE diagnostics [20].

CLIFT utilizes the protist* Crithidia luciliae *as substrate, taking advantage of its kinetoplast, a network of tightly packed dsDNA within a large mitochondrion. In contrast to the nucleus, the kinetoplast contains fewer proteins and thus allows a more selective detection of anti-dsDNA antibodies [21]. Sensitivities of the assay have been reported to range from around 30% to nearly 60% at very high disease specificities of typically above 95% [12, 14–16]. Therefore, CLIFT is appreciated as a useful tool to support the diagnosis of SLE and its discrimination from other diseases.

A limitation of CLIFT however is—as generally applies to the procedure of indirect immunofluorescence tests (IIFT)—the manual read-out of fluorescence signals and its subjective interpretation which lead to a high intra- and interlaboratory variability [12, 22–25]. Great efforts, therefore, have been made in previous years to develop automated solutions enabling optimal image acquisition as well as objective and standardized evaluation of immunofluorescence results, especially in the major field of ANA diagnostics [24, 26, 27]. IIFT on HEp-2 cells still is the recommended “gold standard” for ANA determination [28–30]. Thus, several commercial platforms for automated immunofluorescence microscopy have been developed and validated [31–37]. The automation was shown to greatly contribute to standardization and facilitation of ANA HEp-2 IIFT interpretation. Particularly with regard to positive/negative discrimination, the new systems achieved a very high consensus with manual result interpretation [38–41].

Among these platforms, the EUROPattern-Suite (Euroimmun AG, Lübeck, Germany) is a system for computer-aided immunofluorescence microscopy, combining several hardware and software modules for fully automated image acquisition and evaluation. It performs reliable discrimination of positive and negative ANA HEp-2 (and HEp-20-10) IIFT results. Additionally it provides the option of automated and accurate recognition of several single as well as mixed ANA patterns and titer estimation [32, 39, 40, 42]. Results and corresponding images are displayed within a user-friendly graphical interface (GUI) which allows interactive revision and requires final validation by the professional operator. Thus, the system can reach full compliance with visual immunofluorescence microscopy in terms of result interpretation. In comparison to classical microscopy, the EUROPattern-Suite requires less hands-on effort and is much more resistant to human error. A detailed description of the technology and its associated laboratory management system EUROLabOffice is provided by Krause et al. [42].

Here, we present the first data on automated fluorescence interpretation of CLIFT using the EUROPattern-Suite. Only very few systems have been described in this context in the literature so far [34, 43, 44].

## 2. Material and Methods

### 2.1. Human Sera

A panel of 569 consecutive human sera which were sent to an immunological reference laboratory (Lübeck, Germany) for routine anti-dsDNA screening as well as 100 sera of healthy blood donors were examined. Samples were blinded for the analysis, which was carried out in accordance to the ethical guideline stated in the Declaration of Helsinki (1964).

### 2.2. *Crithidia luciliae* Indirect Immunofluorescence Test (CLIFT)

Indirect immunofluorescence on* Crithidia luciliae* was performed using the Crithidia luciliae (anti-dsDNA) EUROPattern kit following the manufacturer's instruction (Euroimmun AG, Lübeck, Germany). One slide contains 10 reaction areas, each provided with one biochip (2 × 2 mm fragments of coated cover slip glued into the reaction fields), coated with cells of the protist. Slides were manually incubated and washed with the help of the TITERPLANE technique. Samples were applied at a dilution of 1 : 10 in PBS-Tween. Fluorescein isothiocyanate (FITC-) labeled goat anti-human IgG was used for green fluorescent staining. Antiserum was supplied with Evans blue, used for red fluorescent counterstaining of the cells.

### 2.3. Evaluation of Anti-dsDNA Antibodies

A focused image of each biochip on the incubated slides was automatically taken by the EUROPattern fluorescence microscope (see description below). Images were then interpreted in terms of sample positivity/negativity, once automatically by the EUROPattern software and, in a parallel approach, visually by two experts working independently of each other and without notice of software results. Disagreements between visual results were decided by a third opinion. Anti-dsDNA titers of ≥1 : 10 were considered positive.

### 2.4. Description of the System

A detailed description of the general EUROPattern-Suite hardware and software composition is provided in [32, 42].

A new classification software has been specifically developed for the recognition and interpretation of anti-dsDNA on* Crithidia luciliae*: Two images per biochip, one in the green and one in the red fluorescence channel of the microscope, are taken at a 400-fold magnification, using the 40x microscope objective. On average, this magnification leads to the recording of 30 cells per image. Autofocusing is performed using transmitted light to avoid fluorescence bleaching. The underlying algorithm for subsequent fine adjustments has been adapted to the needs of* Crithidia luciliae *image acquisition, resulting in a focused fluorescence image at a resolution of 2,448 × 2,048 pixels within 18 seconds. Thus, a slide containing 10 biochips is processed in less than three minutes. Since the EUROPattern microscope is equipped with two cameras, corresponding images in the green (specific FITC fluorescence signal) and the red (Evans blue counterstaining) fluorescence channel are taken at the same time.

**Figure 1:**
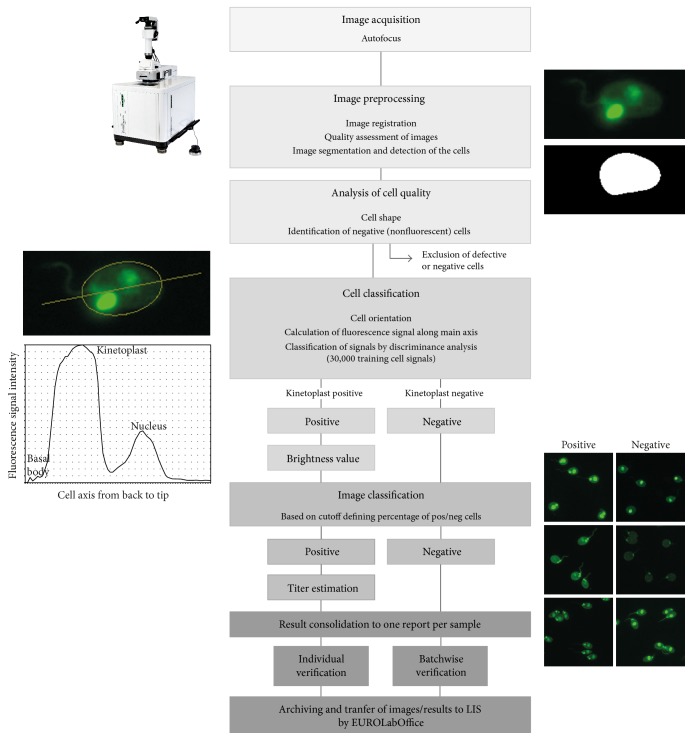
Flowchart of EUROPattern-Suite algorithm for computer-aided immunofluorescence microscopy of* Crithidia luciliae *IFT applied for the detection of antibodies against dsDNA.

The following image classification process operates asynchronously, meaning that the software already provides the first results for interactive verification while the microscope is still running. The procedure incorporates multiple steps which are performed in sequential order (Figure 1).

- *Image Preprocessing*. Images of the green and red channel are loaded to memory and a perfect overlay of each image pair is generated which is important for subsequent image segmentation. Images are then analyzed regarding focal imprecision and potential incubation artefacts by application of specific software algorithms incorporating convolutional filter. Controlling the image sharpness is performed in the red fluorescence channel in which every cell can be detected. This strategy allows reliable discrimination between images of anti-dsDNA negative cells and images taken out of focus. As assessed by an internal visual validation, less than 0.1% of acquired images may reveal some degree of focal inaccuracy. Afterwards, image segmentation and precise detection of any cell are again performed in the red fluorescence channel. Adaptive thresholding techniques are used to mask as well as select every cell by means of connected components.
- *Analyzing Cell Quality*. Each cell mask is now examined in terms of shape characteristics and potential defects, such as size, ellipse form, aspect ratio, or defects of the convex hull. Defective cells are excluded from subsequent evaluation. Additionally, negative cells are identified by application of a threshold-based algorithm which assesses the brightness of the complete cell. If all cells are “dark,” the complete image is set to negative and not processed any further.
- *Cell Classification*. For this purpose, the kinetoplasts of the recognized and fluorescent cells need to be identified. This is achieved by the following steps. First, the software determines the orientation for each cell. Afterwards, a discrete and normalized signal is generated along the calculated main axis, based on mathematical measures such as mean value and standard deviation. Due to the set order of the cellular organelles (basal body, kinetoplast, and nucleus), the signal encodes their specific fluorescence intensities and even discloses the case of an organelle being absent. In a second step, extracted normalized signals are now classified by means of a discriminance analysis based on a reference training database. This database contains images of 30,000 incubated cells which have been acquired from incubations in the reference laboratory or in the context of validation studies. Each of the training images has been labelled by experts with specific information indicating the presence or absence of any fluorescent cell organelle. As a result of this classification, every cell is assigned as either positive or negative according to the fluorescence status of the kinetoplast. Furthermore, a brightness value is extracted from the kinetoplast fluorescence signal.
- *Image Classification*. Images are classified into anti-dsDNA positive or negative, based on a configurable cutoff which defines the required percentage of positive cells within the image. Titer estimation per image is achieved by aggregating the cell brightness values and transforming them into an antibody titer (given in configurable titer steps). A confidence value is calculated which corresponds to the probability of the proposed results. Classifications and titer proposals concerning different dilutions of the same sample are merged into one final result which is given adjacent to the corresponding images within the GUI at the computer screen. The software generated result has to be verified by the physician and confirmed by one mouse click. This verification can be performed batchwise for negative samples and is executed one by one for positive samples.

## 3. Results

**Figure 2:**
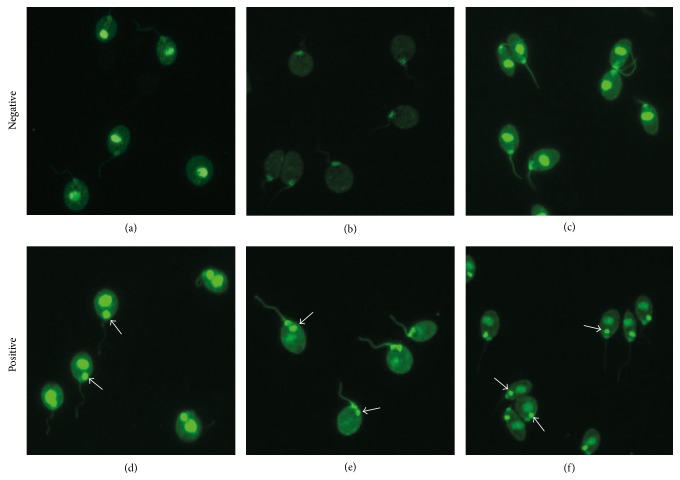
Immunofluorescence patterns on* Crithidia luciliae *revealing the absence or presence of antibodies against dsDNA. Samples are determined as anti-dsDNA negative if the kinetoplasts of the cells do not fluoresce, irrespective of fluorescent nuclei (a), basal bodies (b), or both (c). Samples are determined as anti-dsDNA positive as soon as the kinetoplasts reveal fluorescence signals above a given threshold (see arrows) which may be accompanied by additional fluorescence of the nuclei (d), the basal body (e), or both (f).

Beneficial automation of IIFT evaluation in a diagnostic laboratory requires reliable interpretation of the fluorescent images in clinical routine, encompassing high accuracy of software derived results compared to classical visual inspection by an expert and efficient operation and processing of the system. In case of CLIFT, this means accurate discrimination between positive and negative anti-dsDNA findings according to the presence or absence of a fluorescent* Crithidia luciliae* kinetoplast (Figure 2) within a time- and labor-saving evaluation work flow.

Therefore, the performance of the EUROPattern-Suite (CLIFT classification software) has been validated on the basis of a large number of consecutive sera which have been tested for anti-dsDNA with the help of a commercial CLIFT kit (Euroimmun AG, Lübeck, Germany). Images of all samples were automatically taken by the EUROPattern microscope. Every image revealed a high focal precision, as determined by the software algorithm (see Section 2), thus all of them were suited for subsequent evaluation.

### 3.1. Positive/Negative Classification (All Samples)

Images of 669 tested samples in total were evaluated in terms of positivity/negativity either automatically by EUROPattern software or visually by two experts for CLIFT interpretation (Table 1). Visual inspection yielded 73 anti-dsDNA positive and 596 anti-dsDNA negative sera. Software generated results were 100% accurate with respect to positivity implying that the system likewise recognized the same 73 anti-dsDNA positive samples. Of note, 93% of the EUROPattern titer proposals for positive samples were concordant to visual estimations within the scope of reproducibility of immunofluorescence assays (+/−1 titer level, data not shown). Out of the 596 anti-dsDNA negative samples, the software correctly recognized 577 cases. The remaining 19 samples were determined to be negative by eye but positive by the software. Overall results correlate to a 100% sensitive and 96.8% specific determination of anti-dsDNA on* Crithidia luciliae* by the EUROPattern-Suite. Compared to visual microscopy, overall accuracy of the software was as high as 97.2%.

## 4. Discussion

The need for standardization and automation of IIFT is tremendous in all fields of autoimmune diagnostics in order to ensure objective antibody determination. The technological progress has generated automatic solutions for incubation and processing of slides and, primarily concerning ANA diagnostics on HEp-2 cells, even imaging and evaluation of fluorescence results [39]. Several platforms for IIFT automation, which differ in certain features (e.g., throughput, walk-away times, and DNA counterstain) and applications (e.g., pattern classification, number of recognized patterns, recognition of different substrates, and titer estimation), have been developed and launched for commercial use, facilitating and standardizing ANA IIFT diagnostics in numerous laboratories worldwide [38–42]. Efforts though have been majorly focused on the recognition and interpretation of the HEp-2 cell substrate. Only few commercial systems, among these the EUROPattern-Suite, provide the expanded option of automated evaluation of other substrates such as* Crithidia luciliae *[34, 42], which represents the most important IIFT substrate to detect anti-dsDNA in the context of diagnosing SLE. More comprehensive reports on an automated platform to support* Crithidia luciliae* image classification have been published for a noncommercial computer-aided-diagnosis (CAD) system previously [43, 44].

Similarly to the EUROPattern-Suite technology, the presented CAD system applies a multistep classification approach. Since the optical elements of the EUROPattern microscope, camera resolution, optical magnification, and cell density are adjusted to each other with high-precision, a single image is sufficient for accurate classification of one well/biochip. This leads to high performance and sample throughput in routine diagnostics. The CAD system requires classification of three to five images to classify one well, resulting in one additional classification step compared to the EUROPattern-Suite approach (see Figure 1 in this work and Figure 3 in [43]).

A threshold-based preclassification step is included in both system architectures identifying and separating either negative images (CAD) or negative cells (EUROPattern). The cell-based preclassification of the EUROPattern-Suite is robust regarding image artefacts, avoiding inclusion of false positive images into the further classification process. Single cell classification, based on extracted features, is then applied in the CAD as well as in the EUROPattern system to decide whether single cells and finally the image is positive. However, about 8 cells are recorded per image by the CAD system [44] while the average number of cells within an image taken by the EUROPattern-Suite is about 30.

We did not find any information whether a counterstaining of the cells is used by the CAD system. In the case of the EUROPattern-Suite, Evans blue counterstaining is applied for reliable evaluation of image sharpness and for robust cell segmentation. An option of titer prediction, as provided by EUROPattern, has not been described for the CAD system [43, 44].

Within the scope of this study, the performance of the EUROPattern-Suite in daily clinical routine has been validated. 569 consecutive sera which have been submitted to a reference laboratory for routine anti-dsDNA screening and 100 samples from healthy blood donors were examined by CLIFT to determine anti-dsDNA. Automatically generated results by the EUROPattern-Suite were compared to results obtained through classic visual inspection by two independent experts. This comparison revealed a high accuracy of the automated evaluation strategy. In total, 97.2% of the samples were equally classified. An agreement of 91% was previously reported for another commercial system [34]. Rare cases of inaccuracy in our study exclusively concerned the negative class, as determined by the experts' visual examination, which were proposed to be positive by the software (specificity 96.8%). These false positive classifications were primarily caused by an intensive fluorescence of the basal bodies within the analyzed cells, which was misleading for the software. Further efforts are now focused on the elimination of this inaccuracy. Nevertheless, the physician may rely on the system detecting all positive samples (100% sensitivity), meaning negative results require no closer inspection anymore. Within the GUI, these are displayed in a list, sorted by brightness and classification confidence, and can be easily verified in batches directly at the computer screen. Since the physician's verification is required for generation of the final result, this batchwise processing significantly enhances the overall efficiency of the system. The physician can now focus on the necessary review of the positive samples. Regarding these, the software allows inspection of the images as well as corrections (if required) and validation of the results one after another. Misleading result proposals, therefore, should be counterbalanced by the physician's revision.

The results support the idea of the EUROPattern-Suite standardizing and facilitating SLE diagnostics through automation of CLIFT interpretation. The system is less prone to human interpretation error and functions consistently and time-effectively allowing a long walk-away time [42]. At the same time, it reached concordance with experts of CLIFT interpretation in the vast majority of cases tested during this study. All images and proposed results are displayed within the clearly arranged GUI, which is analogously designed to the ANA evaluation screen and likewise incorporated into the superordinate laboratory management software called EUROLabOffice (ELO) [42]. The results may be revised and need to be validated by the physician in a last step to generate an official end result and a diagnosis directly at the computer screen. Thus, the system has the potential to reach 100% accuracy with respect to visual immunofluorescence microscopy.

Beyond that, ELO manages the complete communication between any standard laboratory information system and the different workstations which can be found in a diagnostic laboratory (e.g., ELISA, IIFT, and immunoblot). All results which have been validated by the physician are reported to ELO and will be merged into one concluding report concerning one sample. The report is integrated into the patient's data history which is accessible via ELO. The integrated database harbors enough memory to additionally save worklists and analytical data, for example, IIFT and immunoblot images. Thus, ELO ensures automated data transfer avoiding errors by manual input, increases laboratory efficiency by taking over several organizational tasks, and supersedes the classical laboratory paper archive.

### 4.1. Perspectives

Further applications of the EUROPattern-Suite are currently under validation or development. These include the already available option of automated immunofluorescence evaluation of antibodies against neutrophil granulocytes (ANCA) using human cell (granulocytes) substrate. The software performs positive/negative classification as well as ANCA pattern discrimination as majorly required in vasculitis diagnostics. Automated documentation and image acquisition of numerous tissues (e.g., liver, kidney, stomach, esophagus, small intestine, heart, and neuronal tissue), which are provided as cryosections on the biochips, is also feasible. With their help, organ as well as nonorgan specific antibodies (e.g., anti-mitochondrial antibodies (AMA), antibodies against epithelial membranes (EMA), epidermal basement membrane or desmosomes, anti-heart muscle-antibodies, or antibodies against various neuronal proteins) may be detected which play an important role in the diagnostics of various other autoimmune diseases.

## 5. Conclusion

Automated determination of antibodies against dsDNA using the EUROPattern-Suite for computer-aided immunofluorescence microscopy on* Crithidia luciliae *represents a new tool in SLE diagnostics. Validation of this system revealed 100% sensitivity and high specificity (96.8%) for recognition and discrimination of anti-dsDNA positive and negative samples compared to visual inspection by experts of CLIFT evaluation. To raise the system's efficiency, negative samples can be verified in batches while positive samples are individually controlled and verified by an expert. In combination with the superordinate laboratory management system EUROLabOffice, the EUROPattern-Suite, therefore, enables a more standardized interpretation of CLIFT and a reduction of laboratory workload.

## Figures and Tables

**Table 1:** Comparison of software-generated and visual positive/negative classification including 669 analyzed samples.

*n* = 669		Visual evaluation		Σ
		Positive	Negative	
EUROPattern	Positive	**73**	*19 *	92
	Negative	*0 *	**577**	577
	Σ	73	596	669

Sensitivity	100%			
Specificity	96.8%			
Accuracy	97.2%			

Concordant results are presented in bold font, differing results in italic font.
